# Assessment of medical students’ integrated clinical communication skills: development of a tailor-made assessment tool

**DOI:** 10.1186/s12909-019-1557-3

**Published:** 2019-04-29

**Authors:** M. Brouwers, J. Custers, E. Bazelmans, C. van Weel, R. Laan, E. van Weel-Baumgarten

**Affiliations:** 1 Radboud Institute of Health Sciences, Dept. Primary and Community Care (161), Radboud University Medical Center, PO Box 9101, 6500 HB Nijmegen, The Netherlands; 2 Departement of Medical Psychology, Radboud University Medical Center, PO Box 9101, 6500 HB Nijmegen, The Netherlands; 3 Department of Health Services Research and Policy, Australian National University, Canberra, Australia; 4 Health Academy, Radboud University Medical Center, PO Box 9101, 6500 HB Nijmegen, The Netherlands

**Keywords:** Clinical communication skills, Communication skills, Physician-patient relations, Assessment, Patient-centeredness, Reliability, Validity, Reproducibility of results

## Abstract

**Background:**

Since patient-centered communication is directly connected to clinical performance, it should be integrated with medical knowledge and clinical skills. Therefore, clinical communication skills should be trained and assessed as an integral part of the student’s clinical performance. We were unable to identify a tool, which helps when assessing patient-centered communication skills as an integrated component of medical history taking (‘the integrated medical interview’). Therefore, we decided to design a new tailor-made assessment tool, the BOCC (BeOordeling Communicatie en Consultvoering (Dutch), Assessment of Communication and Consultation (English) to help raters assess students’ integrated clinical communication skills with the emphasis on patient-centred communication combined with the correct medical content. This is a first initiative to develop such a tool, and this paper describes the first steps in this process.

**Methods:**

We investigated the tool in a group of third-year medical students (*n* = 672) interviewing simulated patients. Internal structure and internal consistency were assessed. Regression analysis was conducted to investigate the relationship between scores on the instrument and general grading. Applicability to another context was tested in a group of fourth-year medical students (*n* = 374).

**Results:**

PCA showed five components (Communication skills, Problem clarification, Specific History, Problem influence and Integration Skills) with various Cronbach’s alpha scores. The component Problem Clarification made the strongest unique contribution to the grade prediction. Applicability was good when investigated in another context.

**Conclusions:**

The BOCC is designed to help raters assess students’ integrated communication skills. It was assessed on internal structure and internal consistency. This tool is the first step in the assessment of the integrated medical interview and a basis for further investigation to reform it into a true measurement instrument on clinical communication skills.

## Background

Doctor-patient communication plays an essential role in providing excellent and safe medical care. Research has shown that 70–80% of medical diagnoses are made by adequate history taking alone [1–3]. In addition, patient-centered communication also leads to improved patient satisfaction and adherence, better health outcomes, such as reduced level of discomfort and worries, and better mental health [4–6]. Fewer diagnostic tests and referrals, indicating an increased efficiency of care, are also shown [7, 8]. Patient centered communication is also related to a reduction in malpractice claims [9].

Communication skills can be acquired and improved through teaching and skills training in students [10] and physicians [11, 12]. As a consequence, clinical communication skills training is now part of the curriculum of a large number of medical schools around the world [13, 14].

Since patient centered communication is directly connected to clinical performance (how the doctor performs overall), clinical communication skills should be integrated with medical knowledge and clinical skills [15, 16]. In other words, it is not only necessary to communicate in a patient-centered manner, but the (future) physician should be able to combine this with the correct medically oriented questions (integrated communication skills or ‘integrated medical interview’). As a consequence, these clinical communication skills should be trained and assessed, preferably as an integral part of students’ clinical performance [5, 15–17].

Assessment of acquired clinical communication skills makes it possible to provide concrete and focussed feedback to learners and drives learning [18–20]. Accordingly, this paper is directed at the assessment of student’s integrated clinical communication skills during their medical training.

Although several instruments to assess communication skills exist [21–23], we were unable to identify any instrument, which assessed patient-centered clinical communication skills as *integrated* components of the medical history taking, suitable for summative assessment of medical students by expert-raters during an OSCE-situation. Instruments such as the Mini-Clinical Evaluation Exercise (Mini-CEX) [24], Integrated Procedural Performance Instrument (IPPI) [25], Maas- Global [26], Frankfurt Observer Communication Checklist (FrOCK) [27] or Communication Assessment Tool (CAT) [28] are designed to assess communication skills, but all of these were not suitable to be used in our situation of assessing patient-centred communication integrated with the correct medical content, since they are not specifically designed for the assessment of the integrated interview based on the biopsychosocial model as we teach our students. Either these instruments are to be used in a different context (IPPI), are too extensive, e.g. measuring more than just problem clarification integrated with specific history (Maas-Global, Mini-CEX), are not specifically patient-centred (FrOCK) or need multiple assessments (CAT). Therefore, we aimed to develop a new tailor-made tool to help raters focus when assessing students’ integrated clinical communication skills with the emphasis on patient-centred communication combined with the correct medical content (problem clarification integrated with specific history) following our educational model. This is a first initiative to develop such a tool, and this paper describes the first steps in this process, including investigating how the tool performed, how it was used by general practitioners and psychologists and how it related to the grades given.

## Methods

### Setting

In 2005, the Radboud University Medical Center Nijmegen started a new medical curriculum with an extensive longitudinal, helical programme on integrated clinical communication skills [29, 30]. Students perceived the teaching as an essential contribution to their clinical performance, and have evaluated this integrated training positively [16]. This included the longitudinal structure throughout the curriculum, with each new training broadening and deepening skills acquired earlier, and the application - in line with the existing evidence on effective communication skills training to physicians (learner-centered, practice-oriented with role-play, feedback and small group discussions) [11]. The basis of the educational model is rooted in the bio psychosocial model of Engel [31] and includes the definition of patient-centeredness by Stewart et al. [30] which consists of 6 interconnection components, e.g. (1) Exploring both the disease and the illness experience; (2) Understanding the whole person; (3) Finding common ground; (4) Incorporating prevention and health promotion; (5) Enhancing the patient-physician relationship; (6) Being realistic.

**Fig. 1:**
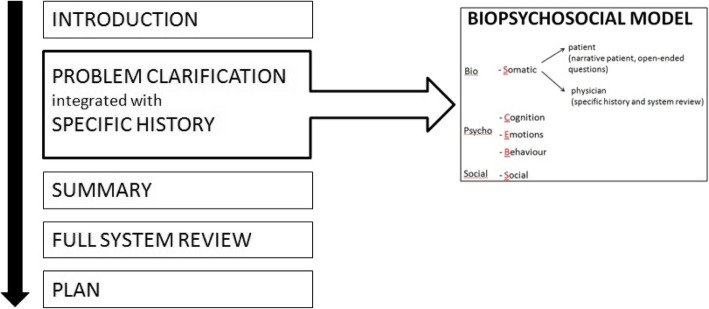
Framework integrated medical interview

Students learn integrated clinical communication skills following the Calgary-Cambridge Consultation Guide (Kurtz, 2005), directed at medical content as well as at five patient centered domains (SCEBS) of the Bio Psycho Social model (see Fig. 1) [16]:

1. Somatic (somatic information about the patient’s problem);
2. Cognition (patient’s ideas and expectations);
3. Emotion (patient’s feelings);
4. Behaviour (how does the problem affect the patient’s behaviour, what does he or she do or avoid because of the problem?),
5. Social (what is the impact of the problem on the social environment, what do significant others perceive, think or do, because of the problem? And how does the patient react to his significant others?)

At the end of the 3rd year of the 6-year medical curriculum, students have a four-week clinical skill course including clinical communication skills training with the involvement of simulated patients (SP). Since the students are at the beginning of learning clinical communication skills, Stewart’s components 1, 2, 3, and 5 are the most relevant at this stage and therefore the basis of the five patient-centered domains of SCEBS.

An essential feature of patient-centeredness of the medical interview as instructed in the curriculum is that in information gathering the students clarify the problem including medical as well as patient perspective. The students learn to apply these domains flexibly by following the patient’s narrative, addressing all domains along the narrative as presented by the patient. But at the same time, the student has to make sure that all five domains are addressed in sufficient depth in relation to the evolving medical information.

In the first, narrative part of the medical interview, the patient is leading, and the student listens actively and asks open-ended and clarifying questions to gather as much information from the patient’s perspective as possible. Ideally, the specific history and the system review questions regarding the problem should be integrated with this ‘problem clarification phase’ by the student, following the patient’s narrative.

This phase is concluded with a summary by the student, including all relevant medical and non-medical information regarding the patient’s problem.

After this, to come to a complete overview of the patient’s general health status, the student should ask all other medical questions in the system review. The medical interview is then concluded with a plan.

This course is concluded with a summative assessment of integrated skills using an Objective Structured Clinical Exam (OSCE) rated by psychologists and general practitioners, consisting of seven stations (two stations communication skills, four stations physical examination, one station clinical reasoning). At the two stations on communication skills, student’s integrated clinical communication skills were assessed, including assessment of exploring with open-ended questions and attentive listening to more closed-ended questions and giving information, directed at medical content as well as at five patient-centered domains. The overall score of the course is determined by the outcome of these ratings as well as the judgement of the tutor who has supervised the student over a longer period of time prior to the OSCE.

These stations consist of two different medical interviews of 9 min each, with different SP’s. One interview was observed by a general practitioner (case of *stomach ache* or *dyspnoea*), the other by a psychologist (*headache, heartburn or dizziness*). The very experienced SP’s were trained extensively for their roles in this OSCE to minimise variation in the role played. The rater team consisted of 13 psychologists (2 males and 11 females) and five general practitioners (all female), who were trained in the use of the tool using training videos with different scenario’s and completing the tool followed by a discussion to optimise inter-rater reliability (one-hour training module).

Students were not aware of the content of the tool, but the items of the tool reflected the goals of the training activities in the four-week course, previous to the OSCE.

### Development of the BOCC-tool (BeOordeling Communicatie en Consultvoering (Dutch), assessment of communication and consultation (English))

The tool had to be able to help the rater in assessing the ‘integrated medical interview’ following our framework (see Fig. 1), had to be suitable for consultations with simulated patients (SPs) and had to be convenient to be used by trained raters in a limited time frame of an OSCE-situation. Items had to mark the successful application of patient centered communication integrated with medical knowledge, as promoted in the teaching instructions.

A panel of expert-teachers in the curriculum (psychologists and general practitioners), using their professional background and clinical experience, selected items that addressed the patient’s perspective, the medical content, integration, general communication skills and patient treatment. After critical appraisal by this group, the tool was ready for testing.

This test-version of the tool consisted of 30 items in total, divided into five subgroups: 1) content of problem clarification, 2) content specific history, 3) structure interview, 4) verbal communication skills, 5) treatment of the patient (see Table 1). In the subgroup ‘Specific history’, four extra items were assessed only in case of pain. Scores ranged from good-moderate-poor-not shown (four-point global rating scale) [32]. A RUBRIC for each scenario explained every item separately. For the subgroup ‘Treatment patient’ only two response categories were used: ‘moderate - poor’. Raters were also asked to give their overall opinion of the students’ communication performance on a scale of 1 to 10, with 10 excellent and 1 the very opposite, further referred to as ‘grading’. *A scoring manual (in Dutch) is available and can be sent by the first author upon request (see email MB).*

**Table 1:** Frequency distribution of the scores of the test- version of the BOCC-tool, *n* = 1344 (in %)

Area	Generated items	Good	Moderate	Poor	Not shown	Not scored
Content of problem clarification	Reason for visit	67.0	31.6	0.3	0.4	0.7
	Somatic information	56.5	38.4	0.3	0.0	4.8
	Cognition	49.3	45.3	1.0	2.2	2.2
	Emotion	33.7	46.1	4.1	13.0	3.1
	Behaviour	45.5	46.2	2.8	2.8	2.8
	Social	49.7	45.8	1.3	1.2	2.0
Content specific history	Main problem	62.7	33.3	0.1	0.1	3.8
	Time start	52.6	42.4	1.2	1.1	2.7
	Evolution in time	52.8	36.7	3.1	2.6	4.9
	Other symptoms	42.3	32.4	5.4	9.0	11.0
	Circumstances that worsen problem	44.5	35.7	2.9	12.9	3.9
	Circumstances that alleviate problem	26.9	38.2	5.5	23.4	5.9
	Effort to relieve problem	50.5	34.9	1.9	10.5	2.2
	System review concerning main problem	31.8	35.5	14.1	14.7	3.8
Structure interview	Introduces him/herself	72.8	24.3	0.9	1.3	0.7
	Summary	33.7	41.2	5.7	15.0	4.4
	Integrates problem clarification with specific history	40.0	46.7	3.2	2.4	7.7
Verbal communication skills	Follows patient	58.7	36.7	1.1	0.1	3.4
	Reflects on emotions	46.9	42.8	4.7	1.7	3.9
	Directs patient	46.1	43.9	1.3	0.3	8.4
	Tests own ideas	44.6	43.7	0.7	2.2	8.8
	Concretizing questions	51.9	40.6	3.0	0.1	4.4
	Responds adequate	48.8	39.7	2.7	0.0	8.9
		Moderate		Poor		
Treatment of patient	External presentation	93.8		0.8		5.4
	Facial expression	94.9		1.5		3.6
	Paraverbal features	90.8		0.7		8.5
	Spatial proximity	91.0		0.7		8.3
	Looks at patient	95.5		0.9		3.6
	Body position	93.8		1.2		5.0
	Body movement and gestures	91.1		1.0		7.8

### Validity and internal consistency of the BOCC-tool

The validity and internal consistency of the tool were assessed based on the students’ results during the OSCE.

As the OSCE was compulsory all third-year medical students of two consecutive years of the medical school (315 in 2009–2010; 357 in 2010–2011, in total 672) were assessed with the help of the tool, who each performed the two OSCE stations, resulting in 1344 tool scores available for analysis.

#### Validity evidence based on internal structure [33]

Frequency distributions of the items were inspected. Next, principal component analysis (PCA) with oblique rotation was performed to determine whether some items clustered together in a subgroup.

#### Evidence based on internal consistency

To assess homogeneity (i.e. do the items measure the same skills necessary for that specific subgroup) of the items within subgroups, Cronbach’s alpha was calculated as we assumed that some subgroups were heterogeneous (i.e. the items measure different skills necessary to meet the standards of that specific subgroup fully. The items together in a group constitute the full skill set that can be shown by the student to meet the standards of that specific subgroup) by nature.

#### Relation between tool scores and grading

Descriptive statistics on the grades were performed. Regression analyses were conducted to assess the explained variance of the subgroups on the grades. This analysis was based on the rater’s general score (grade) of the clinical communication performance of the student during the OSCE. This form of construct validity compared the two different constructs that are assessed, i.e. tool scores and the grading.

#### Applicability

After establishing part of the validity of the tool in the target population (OSCE for 3rd-year medical students with experienced raters), the validity was further tested by investigating the applicability of the tool within a different group of raters and students. (I.e. Can the tool be used in other teaching environments with other raters and still produce the same five components?). Therefore, the tool was used for peer-assessment in a group of 374 4th-year medical students during the regular communication skills training with simulated patients, 9 months after the previously mentioned OSCE. PCA with oblique rotation was performed to determine whether the same five components would emerge.

All statistical analyses were performed with SPSS version 16. Available case analysis with list-wise deletion per analysis in case of missing items was performed.

## Results

### Validity evidence based on internal structure

Table 1 shows that frequency distribution of the scoring categories ‘good’ and ‘moderate’ of the test-version of the BOCC are high for most items. For the subgroup ‘Treatment patient’ almost all students scored the same category ‘moderate’. Therefore this subgroup was not taken into account in the subsequent statistical analysis. Missing data are shown in Table 1.

The Kaiser-Meyer-Olkin analysis yielded an index of 0.89 and Bartlett’s test of Sphericity was highly significant χ^2^ (253) = 5637, *p* < .001, indicating that the distribution of the data met the psychometric criteria for PCA with oblique rotation. PCA analyses of the scores showed an interpretable solution of five components (47.35% of the total variance explained). For reasons of clarity, these 5 components were renamed into subgroups for the final version of the tool: problem clarification (PC), communication skills (CS), specific history (SH), Problem influence (PI), and Integration skills (IS). Analysis showed that both items ‘Behaviour’ and ‘Social’ statistically were assigned to CS. However, based on the loadings it was also possible to maintain them in the original component PC, which better fitted the educational model since the subgroup PC reflects on the content of narrative of the patient following SCEBS as previously mentioned. The item ‘Other symptoms’ did not fit one of the components and was removed from the subsequent analyses. Table 2 displays the five components, the accompanying loadings and communalities of each item.

**Table 2:** Factor Pattern Matrix and Communalities of the test-version of the BOCC-tool

	Factors with loadings					Communalities extraction
	CS	PI	IS	SH	PC	
Responds adequate	.713					.529
Follows patient	.657					.592
Tests own ideas	.644					.618
Concretizing questions	.624					.571
Directs patient	.607					.576
Reflects on emotions	.561					.383
Behaviour	.515					.403
Social	.461					.446
Circumstances worsen problem		.796				.652
Circumstances alleviate problem		.795				.647
Effort to relieve problem		.346				.349
System review main problem			.660			.471
Summary			.646			.462
Integration			.454			.483
Time start				.799		.630
Evolution in time				.701		.472
Main problem				.570		.597
Somatic information				.446		.551
Introduction				.340		.279
Cognition					.696	.536
Emotion					.588	.462
Reason visit					.516	.504

*CS* = Communication skills; *PI* = Problem influence; *IS* = Integration skills; *SH* = Specific history; *PC* = Problem clarification

Since not all cases concerned pain, the items about pain were excluded from the analysis and the further development of the tool. Based on the loadings the items were rearranged, resulting in the final BOCC-tool (Table 3).

**Table 3:** Final version of the BOCC-tool

Problem clarification	Good	Mode-Rate	Poor	Not Shown
Reason for visit				
Cognition (attribution, expectations)				
Emotion				
Behaviour				
Social				
Specific history				
Introduction him/herself				
Somatic information				
Main problem				
Time start				
Evolution in time				
Other symptoms				
Influence problem				
Circumstances that worsen problem				
Circumstances that alleviate problem				
Effort to relieve problem				
Communication skills				
Follows patient (open-ended questions, exploration, affirmation)				
Dealing with emotions: reflection, paraphrases, uses silence				
Directs patient (closed-ended questions, multiple choice questions)				
Tests own ideas (closed-ended questions, multiple choice questions)				
Asks concretizing questions				
Responds adequate: non-suggestive, rhetoric, multiple, yes-buts, medical jargon				
Integration skills				
Gives summary at end				
Integrates problem clarification with specific history				
Finishes system review main problem				
Treatment patient	MODERATE		POOR	
Judgement on treatment patient				
ONLY FILL OUT WHEN TREATMENT NEEDS ATTENTION				
External presentation conform behavioural code				
Facial expression				
Paraverbal features*				
Spatial proximity/distance, touch				
Looks at patient				
Body position				
Body movement and gestures				
Final Grade (1--10)				

*tone of voice, intonation, speed of speaking, pauses

### Evidence based on internal consistency

Cronbach’s alpha of the various subgroups shows values ranging from .42 to .80.

These scores and mean inter-item correlations for the subgroups are displayed in Table 4.

**Table 4:** Internal consistency of the subscales and regression coefficients of the BOCC-tool

Subscale	Internal consistency		Regression Coefficients for predicting grade (Beta β)	
	Cronbach’s alpha α	Mean inter-item correlation	In OSCE (*n* = 959)	In 4th year medical students (*n* = 324)
Communication skills (*n* = 1160)	.80	.41	.206*	.329*
Problem influence (*n* = 1234)	.49	.24	.201*	.075
Integration skills (*n* = 1169))	.42	.20	.246*	.073
Specific History (*n* = 1215)	.70	.33	.015	.101
Problem clarification (*n* = 1263)	.49	.18	.259*	.202*
			R Square .351	R Square .351

* *p* < .001

### Relation tool scores and grading

The mean grade given was 7.15 (SD 0.76, range 5.0–9.0). Standard multiple regression was used to assess the ability of five subgroups to predict the grading. To compare the contribution of each subgroup, the sum scores of each subgroup were calculated to determine the beta values. As shown in Tables 4, 35% of the overall variance in grading is explained by the subgroups. The subgroup ‘Problem clarification’ made the strongest unique contribution to the prediction of the grading, when the variance explained by all other variables in the model is controlled for. Three other subgroups were making a smaller, but statistically significant, unique contribution to the prediction of the grading (Communication skills, Problem influence, Integration skills). The subgroup ‘Specific History’ did not add a significant unique contribution to the prediction of the final grading of the rater. Regression coefficients for the subgroups are displayed in Table 4.

### Applicability

Three hundred seventy-four completed tools were analysed by explorative PCA analysis to investigate applicability to another context (other raters and students). This showed that the found factors were similar to those found during the OSCE-exam (not displayed).

Multiple regression was used to identify whether within this context the subgroups were also able to predict the general grading of the raters. The subgroup ‘Communication skills’ had the largest influence on prediction, next to the subgroup ‘Problem clarification’. Subgroups ‘Specific history’, ‘Problem influence’ and ‘Integration skills’ did not contribute significantly to the general grading (see Table 4).

## Discussion

This paper reports on the development of a tool, the BOCC, as an assessment help on integrated communication skills. It was aimed to help raters to assess patient centered communication skills *integrated* with the application of medical knowledge and clinical skills since both are vital to adequate clinical performance. The tool was intended to be used in an OSCE-situation by guiding the rater based on the used educational theory on the integrated medical interview, and we provided validity evidence based on internal structure and consistency. Furthermore, it was shown that the subgroup ‘Problem clarification’ (Cognition, Emotion and Reason for visit) contributed most to the prediction of the grading of students.

Research suggests that OSCE’s are suitable for high-stakes assessments, can be used for assessment of clinical skills and complex communication skills and seem the most appropriate assessment to predict future clinical performance [34–36]. Besides several general measurement tools on communication skills, such as the Maas-Global and Four Habits [26, 37, 38], specific rating scales on communication skills designed for OSCE’s are also available (e.g. ‘Explanation and Planning Scale (EPSCALE) and Common Ground (CG) and should be part of a more extensive assessment program (programmatic assessment) which uses multiple data points (over time) to come to a conclusion about student’s skills [39]. This programmatic assessment is more and more considered to be state of the art in assessment.

However, as pointed out by Setyonugroho et al. in their review on OSCE checklists on communication skills, there is no consensus in the interpretation and definition on domains of communication skills, and consequently desirable performance standards, across the world [35]. Comert et al. [40] support this, demonstrating that most rating scales use different definitions of communication skills. Furthermore, it is shown that most studies on rating scales in OSCE’s have a poor methodological quality regarding reliability and validity issues (including content validity) [40]. Hodges showed that some individual rating scales are sensitive to the level of training [32], and should be carefully selected when designing a tool for assessment of communication skills.

Adequate clinical communication skills consist of patient-centered communication integrated with adequate medical knowledge and clinical skills. However, none of the above described instruments assesses the integration of the medical interview [41]. The BOCC-tool is a first attempt to help integrated assessment of both patient-centered communication skills and general communication skills, with medical content. It consists of five components, reflecting the framework on the integrated medical interview as previously outlined.

These five components represent the five subgroups of the BOCC-tool with different internal consistency scores. Since the items in each subgroup represent different skills, that not necessarily are linked but instead represent the full range of skills of that specific subgroup, Cronbach’s alpha for some components is below .50. Each item in the subgroup measures a different aspect of the subject in the subgroup and therefore cannot be internally consistent. The rater is given the opportunity to rate the subgroup by using the individual items to come to a conclusion about that specific aspect. Therefore, the Cronbach’s alpha value should not be seen as a measure of reliability, but rather as an explorative description of the subgroup. Although some subgroups show a low score, we believe that the items in the subgroup belong together and represent the heterogeneity within that specific subgroup. Further research is needed on the low internal consistency that might be co-determined by students ‘task-specific focus’ on their performance or their individual behaviours and work style. Test-retest reliability and inter-rater reliability should be investigated to come to a further understanding of the tool’s reliability.

The assessment is concluded with the general grade by the experienced professional (expert-rater) on student’s overall communication skills. As mentioned by Huntley et al. [40] “Communication is (...) inherently creative and cannot be developed simply by learning skills and rules for combining and displaying them.” They stress that “raters should make aesthetic judgements about whether communication ‘works.” This general grade should reflect the holistic impression of the interview by the expert-rater, preventing unjust high OSCE scores in the situation in which a student follows all the rules of communication, but that the overall impression of the medical interview is awkward.

An interesting finding is, that the component Problem clarification (with items concerning Cognition, Emotions and Reason for visit) contributes strongest to the general score (grade) by the rater, with the components Communication Skills, Problem Influence and Integration Skills coming second. This is consistent with our experience during teaching this course, in which students say that they find it most difficult to talk about patient’s cognitions and emotions. To do this naturally and following the patient’s narrative demands elegance and creativity in the student’s communication skills to achieve the best result. Students often easily settle for less on these subjects due to discomfort and quickly go on with the medical content, resulting in lower general grades. Our study indicates that when a student can explore in depth the cognitions and emotions from the patient’s narrative, this is reflected in a higher overall grade by the expert-rater, who apparently values this most important.

However, in the group of 4th-year medical students, which assessed their peers, the score on the subgroup ‘Communication Skills’ best predicted the general grade. This subgroup mostly consists of observable discrete behaviours such as following patient, concretising questions, and reflection on emotions. Of course, novice communicators will be more primed to these behaviours, since this is what they learned and consequently reward this with higher grades.

Although the BOCC-tool is designed to be used in an OSCE, we think it might also be useful for formative feedback during communication skills training, since its generalizability is proven sufficient in the context of another group of medical students. The BOCC can provide direct feedback on both communication skills and medical content with limited time investment of the observers and gives learners the opportunity to try alternative behaviour based on its detailed information.

Furthermore, the BOCC might be useful to single out the non-function student, who is not able to establish adequate contact in the observed interview. Since the subgroup ‘Treatment patients’ has a skewed distribution (see Table 1), and apparently does not discriminate enough between either good or bad students, we decided not to include this subgroup in the subsequent analysis. However, we maintained it in the BOCC-tool to provide the expert-raters with an opportunity to signal poor performance on this domain.

It has two scoring options for the item addressing ‘Treatment patient’, based on our assumption that the behaviour reflecting this item is dichotomous in nature: either the observed behaviour is adequate for the interview or is not adequate. However, since the described items are very much subjective to the observer, a caveat which concerns the use for this purpose is in place.

Our study has several limitations. First, this is the first evaluation of a new tailor-made tool. Further independent investigation of the tool on reliability and validity properties is necessary to enhance it. For example, when this tool is to be used to assess students in time (longitudinal) inter-rater and test-retest reliability, responsiveness and interpretability should be investigated.

Second, the large sample of students is rated by various general practitioners and psychologists. To investigate inter-rater reliability, it would be necessary to investigate these rater groups separately. In addition to this, it would be fascinating to investigate the relationship between the rater and the grading and study if the rater’s background (physicians vs. psychologists) influences the grading of students when using the same context. For the future the instrument might be adjusted for use by other health professions who also use OSCE’s as part of their training and assessment. This could facilitate an interprofessional basis in the education of health professionals.

## Conclusions

This study provides a first investigation of a newly developed tailor-made tool to help raters in assessing integrated communication skills in an OSCE situation of 3rd-year medical students. We have described our view on the integrated medical interview and provided validity evidence based internal structure, consistency and applicability. The advantages of this new tool include that it is based on an educational model of the integrated interview; it guides raters when assessing communication skills during an OSCE and indicates on what domains a student lacks skills. Disadvantages include the fullness of detail, the lack of a numeric (total) score of the tool and unknown reliability including inter rater reliability and test-retest reliability. Of course, additional investigation is necessary to explore the reliability and validity properties of the BOCC-tool further and provide more insight into the possibilities of transforming the tool into a true measurement instrument. In conclusion, this tool is a first step in the integrated assessment of the integrated medical interview.

## Abbreviations

BOCC: BeOordeling Communicatie en Consultvoering (Dutch), Assessment of Communication and Consultation (English)
CAT: Communication Assessment Tool
CG: Common Ground
EPScale: Explanation and Planning Scale
FrOCK: Frankfurt Observer Communication Checklist
IPPI: Integrated Procedural Performance Instrument
Mini-CEX: Mini-Clinical Evaluation Exercise
OSCE: Objective Structured Clinical Exam
PCA: principal component analysis
SCEBS: Somatic-Cognition-Emotions-Behaviour-Social domains
SP: simulated patient
